# A multi-sensor wearable system for the assessment of diseased gait in real-world conditions

**DOI:** 10.3389/fbioe.2023.1143248

**Published:** 2023-04-21

**Authors:** Francesca Salis, Stefano Bertuletti, Tecla Bonci, Marco Caruso, Kirsty Scott, Lisa Alcock, Ellen Buckley, Eran Gazit, Clint Hansen, Lars Schwickert, Kamiar Aminian, Clemens Becker, Philip Brown, Anne-Elie Carsin, Brian Caulfield, Lorenzo Chiari, Ilaria D’Ascanio, Silvia Del Din, Bjoern M. Eskofier, Judith Garcia-Aymerich, Jeffrey M. Hausdorff, Emily C. Hume, Cameron Kirk, Felix Kluge, Sarah Koch, Arne Kuederle, Walter Maetzler, Encarna M. Micó-Amigo, Arne Mueller, Isabel Neatrour, Anisoara Paraschiv-Ionescu, Luca Palmerini, Alison J. Yarnall, Lynn Rochester, Basil Sharrack, David Singleton, Beatrix Vereijken, Ioannis Vogiatzis, Ugo Della Croce, Claudia Mazzà, Andrea Cereatti, for the Mobilise-D consortium

**Affiliations:** ^1^ Department of Biomedical Sciences, University of Sassari, Sassari, Italy; ^2^ Interuniversity Centre of Bioengineering of the Human Neuromusculoskeletal System (IuC BoHNes), Sassari, Italy; ^3^ Department of Mechanical Engineering, Insigneo Institute for In Silico Medicine, The University of Sheffield, Sheffield, United Kingdom; ^4^ Department of Electronics and Telecommunications, Politecnico Di Torino, Torino, Italy; ^5^ Translational and Clinical Research Institute, Faculty of Medical Sciences, Newcastle University, Newcastle Upon Tyne, United Kingdom; ^6^ National Institute for Health and Care Research (NIHR) Newcastle Biomedical Research Centre (BRC), Newcastle University, Newcastle Upon Tyne, United Kingdom; ^7^ Centre for the Study of Movement, Cognition and Mobility, Neurological Institute, Tel Aviv Sourasky Medical Centre, Tel Aviv, Israel; ^8^ Department of Neurology, University Medical Centre Schleswig-Holstein Campus Kiel and Kiel University, Kiel, Germany; ^9^ Department for Geriatric Rehabilitation, Robert-Bosch-Hospital, Stuttgart, Germany; ^10^ Laboratory of Movement Analysis and Measurement, Ecole Polytechnique Federale de Lausanne, Lausanne, Switzerland; ^11^ Newcastle upon Tyne Hospitals NHS Foundation Trust, Newcastle Upon Tyne, United Kingdom; ^12^ Instituto de Salud Global Barcelona, Barcelona Institute for Global Health (ISGlobal), Barcelona, Spain; ^13^ Faculty of Health and Life Sciences, Universitat Pompeu Fabra, Barcelona, Spain; ^14^ CIBER Epidemiología y Salud Pública, Madrid, Spain; ^15^ Insight Centre for Data Analytics, University College Dublin, Dublin, Ireland; ^16^ Department of Electrical, Electronic and Information Engineering “Guglielmo Marconi”, University of Bologna, Bologna, Italy; ^17^ Health Sciences and Technologies-Interdepartmental Centre for Industrial Research (CIRI-SDV), University of Bologna, Bologna, Italy; ^18^ Machine Learning and Data Analytics Lab, Department Artificial Intelligence in Biomedical Engineering, Friedrich-Alexander-Universität Erlangen-Nürnberg, Erlangen, Germany; ^19^ Department of Sport, Exercise and Rehabilitation, Faculty of Health and Life Sciences, Northumbria University, Northumbia, United Kingdom; ^20^ Novartis Institutes of Biomedical Research, Novartis Pharma AG, Basel, Switzerland; ^21^ Department of Neuroscience and Sheffield NIHR Translational Neuroscience BRC, Sheffield Teaching Hospitals NHS Foundation Trust, Sheffield, United Kingdom; ^22^ Department of Neuromedicine and Movement Science, Norwegian University of Science and Technology, Trondheim, Norway

**Keywords:** gait analysis, IMU, wearable sensors, ecological conditions, pressure insoles, distance sensors, spatial-temporal gait parameters

## Abstract

**Introduction:** Accurately assessing people’s gait, especially in real-world conditions and in case of impaired mobility, is still a challenge due to intrinsic and extrinsic factors resulting in gait complexity. To improve the estimation of gait-related digital mobility outcomes (DMOs) in real-world scenarios, this study presents a wearable multi-sensor system (INDIP), integrating complementary sensing approaches (two plantar pressure insoles, three inertial units and two distance sensors).

**Methods:** The INDIP technical validity was assessed against stereophotogrammetry during a laboratory experimental protocol comprising structured tests (including continuous curvilinear and rectilinear walking and steps) and a simulation of daily-life activities (including intermittent gait and short walking bouts). To evaluate its performance on various gait patterns, data were collected on 128 participants from seven cohorts: healthy young and older adults, patients with Parkinson’s disease, multiple sclerosis, chronic obstructive pulmonary disease, congestive heart failure, and proximal femur fracture. Moreover, INDIP usability was evaluated by recording 2.5-h of real-world unsupervised activity.

**Results and discussion:** Excellent absolute agreement (ICC >0.95) and very limited mean absolute errors were observed for all cohorts and digital mobility outcomes (cadence ≤0.61 steps/min, stride length ≤0.02 m, walking speed ≤0.02 m/s) in the structured tests. Larger, but limited, errors were observed during the daily-life simulation (cadence 2.72–4.87 steps/min, stride length 0.04–0.06 m, walking speed 0.03–0.05 m/s). Neither major technical nor usability issues were declared during the 2.5-h acquisitions. Therefore, the INDIP system can be considered a valid and feasible solution to collect reference data for analyzing gait in real-world conditions.

## 1 Introduction

It is well established that gait impairments affect one’s functional status and overall health, (Laudani et al., 2013; Polhemus et al., 2021), and that a holistic model of functioning and disability should not rely only on a conventional laboratory assessment. Rather, it should also include a quantitative description of a person’s mobility in its own ecological environment to include social and personal factors (World Health Organization, 2001; Giannouli et al., 2016; Galperin et al., 2019; Hillel et al., 2019). Nonetheless, the description of gait in real-world conditions is still a major challenge in people with impaired mobility due to the increased gait complexity associated with changes in speed and direction of progression, slow walking and use of walking aids, presence of breaks, short walking bouts, and confounding factors such as non-walking activities (Mobilise-D 2019). Several technologies and algorithms have been proposed to extract clinically meaningful spatial-temporal digital mobility outcomes (DMOs) across a large spectrum of gait disorders, but technical validity was in most of the cases assessed in a supervised laboratory setting evaluating basic gait tasks (Zijlstra and Hof, 2003; Wang et al., 2016; Pacini et al., 2018; Bertuletti et al., 2019). Further efforts are hence required to generalize results under real-world conditions.

One of the most promising solutions for mobility assessment in ecological conditions is the use of wearable inertial measurement units (IMUs). A single-IMU approach is preferred when maximizing user acceptance is key (Bonci et al., 2020; Mobbs et al., 2022). Conversely, a bilateral lower extremities positioning (i.e., IMUs attached to the shanks or feet) is suggested to obtain a more accurate gait description in people with severe gait disorders (Yang et al., 2013; Bourgeois et al., 2014; Hundza et al., 2014; Trojaniello et al., 2014). However, when using these devices, the identification of gait events (i.e., initial and final foot contact timings), which is a prerequisite for the estimation of the temporal and spatial parameters, is indirectly derived from the linear acceleration and angular velocity signals which vary their morphology, amplitude, and repeatability, depending on specific walking patterns. This implies that the technical validity of the DMOs provided by IMU-based methods should be tested against reference data under the same conditions of end-use. Furthermore, the availability of reliable reference gait data is also essential for the development, optimization, and testing of newly proposed IMU-based machine learning methods (Martindale et al., 2019; Roth et al., 2021a).

A commonly employed solution to obtain a reference for gait detection and activity discrimination is the use of body-worn cameras pointing to the subjects feet (Buso et al., 2015; Full et al., 2015; Hickey et al., 2016). However, besides potential privacy issues, the temporal resolution of this approach depends on camera frame rate. Furthermore, it requires extensive manual intervention for labeling gait events, and it doesn’t provide information on spatial gait parameters nor on turning maneuvers. Conversely, methods based on the use of global navigation satellite systems can potentially provide low positional errors (Terrier et al., 2000), but their performance greatly depends on environmental conditions (Reggi et al., 2022), they aren’t reliable indoor, are characterized by a low temporal resolution, and don’t allow for a stride-by-stride gait description (Atrsaei et al., 2021). An accurate and reliable solution for gait events detection is to use plantar pressure insoles (Hausdorff et al., 1995; Storm, Buckley, and Mazzà, 2016; Roth et al., 2018) as this technology provides a direct sensing of the foot-ground forces (Salis et al., 2021a). When using these systems, however, no spatial information is provided.

To overcome the intrinsic technological limitations of the aforementioned systems, the simultaneous integration of complementary sensing approaches and the exploitation of data redundancy to improve methods employed and optimize performance may be beneficial. In this regard, several research and consumer-grade systems integrating pressure insoles with IMUs attached to the feet have been proposed (Salis et al., 2021b; Duong et al., 2022; Refai et al., 2018; Feetme Devices, 2022b; NURVV, 2022). Based on this sensor configuration, Duong and colleagues (Duong et al., 2022) have proposed a machine learning model for spatial-temporal gait analysis (SportSole II). The method’s accuracy was validated in terms of mean absolute percentage errors on eleven healthy young adults during simple straight and curvilinear walking, whereas ecological validation was performed in terms of DMOs agreement between spatial-temporal parameters estimated in laboratory and real-world conditions. Although the results of this study were promising, with errors of stride length ∼3.5%, the restriction of including only healthy young adults does not support applicability of the systems use within pathological cohorts with potentially impaired gait. In the latest years, several consumer grade systems such as FeetMe^®^ and NURVV^®^ have been made available for healthcare applications (Feetme Devices, 2022b; NURVV, 2022). In general, these commercial systems were designed to improve user-friendliness and provide a full gait report, however, they operate as a black box system whereby the algorithms employed are not described in detail, and their validation procedures are limited to basic gait tasks such as straight walking (Feetme Devices, 2022a).

The aim of this study is thus to present and characterize the performance of a novel multi-sensor system for gait assessment to be employed as reference in people with impaired mobility in real-world. The INDIP system (INertial module with DIstance sensors and Pressure insoles) integrates two plantar pressure insoles for a direct measure of foot-to-ground contacts, three IMUs attached to both feet and lower back for activity recognition, turning detection, and displacement estimation, two time-of-flight infrared distance sensors to detect the alternating movements of the lower extremities.

To meet the emerging demands associated with reproducibility and replicability in biomedical research and regulatory qualification (Viceconti et al., 2020), a complete description of INDIP system hardware specifications and of the algorithms used for DMOs estimation based on standardized operational definitions (Kluge et al., 2021) is provided here. Furthermore, to assess the INDIP performances under testing conditions resembling those likely to be encountered in real life, a multi-task experimental protocol in a lab setting, which included speed and trajectory changes, surfaces and inclinations, obstacles, breaks, and even cognitive demand levels (Mazzà et al., 2021; Scott et al., 2022), was implemented. To evaluate the potential influence of different gait types on the accuracy of the estimated DMOs, gait data of 128 participants were analyzed, including healthy young and older adults, people with Parkinson’s disease (PD), multiple sclerosis (MS), chronic obstructive pulmonary disease (COPD), congestive heart failure (CHF) and proximal femoral fracture (PFF). Finally, INDIP usability was evaluated by recording 2.5 h of unsupervised activity performed in the participants habitual environment in five different clinical centers participating in the IMI2-JU-funded Mobilise-D project (Number 820820) (Mobilise-D 2019; Mazzà et al., 2021).

## 2 Materials and methods

### 2.1 The INDIP system

**FIGURE 1:**
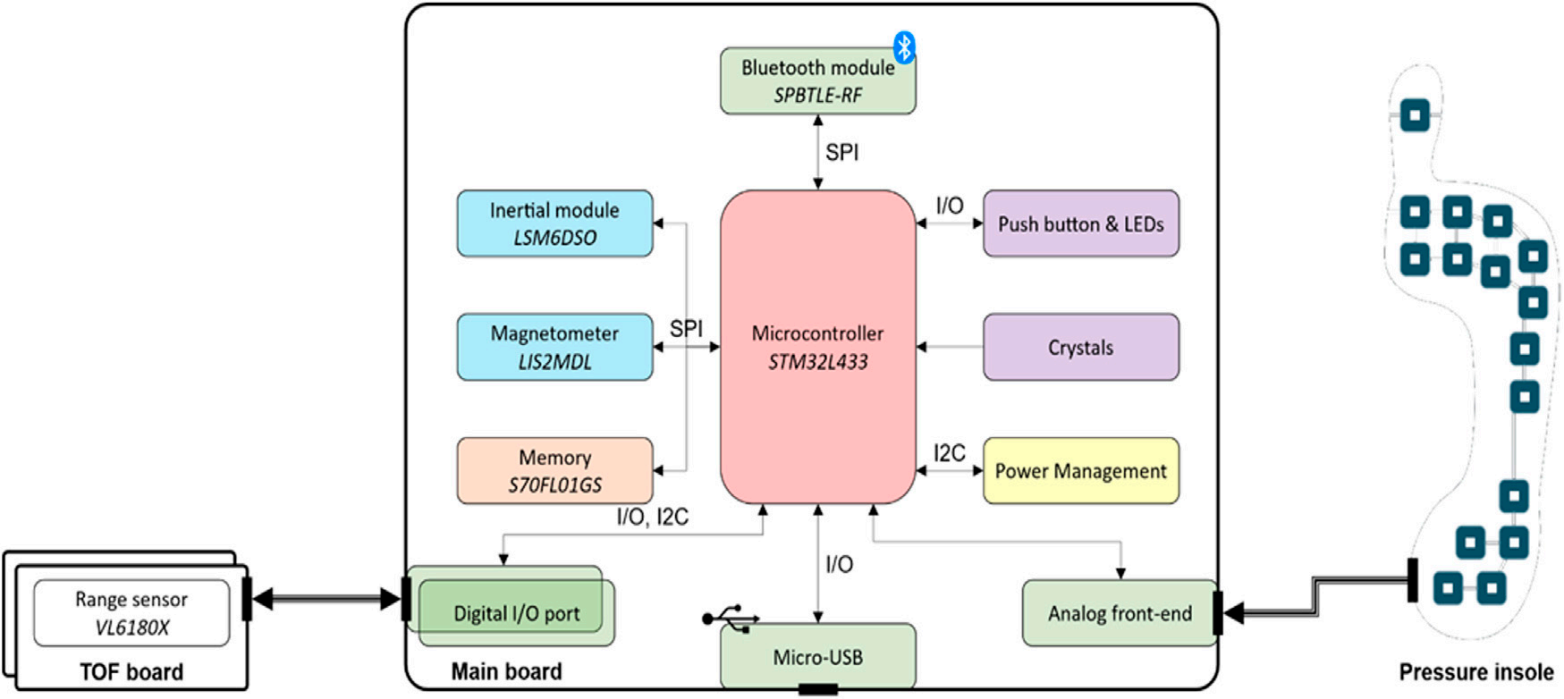
INDIP system architecture which includes the following components: Bluetooth Low Energy module SPBTLE-RF (mfr. STMicroelectronics), inertial module LSM6DSO (mfr. STMicroelectronics), magnetic module LIS2MDL (mfr. STMicroelectronics), memory (S70FL01GS, mfr. Infineon; up to 13 h of data logging), microcontroller STM32L433 (mfr. STMicroelectronics, ARM^®^ Cortex®-M4 32-bit architecture), range sensor VL6180X sensor (mfr. STMicroelectronics).

The central unit of the INDIP system (manufacturer (mfr.) 221e S.r.l. (221 e S.r.l, 2020) is a state-of-the-art magneto-IMU that can be connected to various sensing peripherals. The overall system hardware architecture, as well as the communication interfaces used, is shown in Figure 1. A description of the firmware’s architecture, together with some additional details on the hardware, are reported in Appendix A.

#### 2.1.1 Main board

The main board has been designed to sense motion and process relevant data with a low power consumption, to store recorded data on-board and to offer a wired/wireless transmission. Motion data include both inertial and magnetic data. The inertial module is a system-in-package featuring a 3D digital accelerometer and a 3D digital gyroscope (full-scale ranges set to ±16 g and ±2,000 dps respectively for this study). The magnetic module is an ultra-low-power, high performance 3-axis digital magnetic sensor (magnetic field dynamic range of ±50 G).

One 18-pin (i.e., analog front end) and two 6-pin connectors (i.e., digital I/O port) are mounted on the bottom and right/left side of the main board, respectively (Figure 1). The 18-pin ZIF-connector enables the connection between the pressure insoles and the microcontroller unit through the analog front-end, while the two 6-pin connectors allow the main board to manage any digital sensor that supports the I2C communication protocol (e.g., the distance sensor). The main board acts as a “motherboard,” i.e., supplying the required power and providing storage and connectivity capabilities. Therefore, any external sensing peripheral (e.g., distance sensor, pressure insole) could be designed with the strictly necessary components, thus minimizing its form factor.

An external crystal with a frequency stability of ±5 ppm (parts per million) has been selected to generate more accurate and precise time values. The main board also supports the synchronization with an external equipment in two modes:

• output synchronization: when the main board starts recording data, it outputs a signal to external equipment by exploiting the ID pin of the micro-USB;
• input synchronization: when the main board receives a signal from external equipment on the ID pin of the micro-USB, it starts recording. Input and output signals can be either rising edge or level triggered.

#### 2.1.2 Sensing peripherals

• The Time-of-Flight infrared distance sensor includes an infrared emitter, a range sensor (range set to 0.2 m at 50 Hz for this study), and an ambient light sensor in a three-in-one package. A fully comprehensive characterization while considering different factors, such as target color, sensor-target distance, and sensor-target angle of incidence in both static and dynamic conditions, can be found in (Bertuletti et al., 2017).
• The force sensitive resistor pressure insole (PI) consists of 16 force sensing resistors, with an overall thickness of 240 μm, covered with a polyester layer. Each force sensing resistor exhibits a resistance value which is inversely proportional to the amount of the applied force and, when no force is applied, the sensor features an infinite resistance. As the applied force increases, the equivalent resistance of the sensor decreases. In this study, two different sizes have been used, one small (EU 36–37) and one large (EU 42–43).

### 2.2 Calibration refinement and characterization of the inertial sensor noise level

As sensor performance may deteriorate over time, regular refinements of the accelerometer and gyroscope calibration coefficients are recommended to compensate for residual cross-axis sensitivity and misalignments (Ferraris et al., 1995) (systematic errors). This is beneficial for ensuring good quality of the measurements and facilitating results comparability in multi-center validations. The calibration refinement of both accelerometers (Ferraris et al., 1995) and gyroscopes (Stančin and Tomažič, 2014) was carried out for each of the 72 INDIP IMUs deployed in this study, before their first use. Furthermore, each INDIP IMU was characterized in terms of noise level (random errors). This information was relevant for the optimal tuning of algorithm parameters to estimate orientation and displacement (Caruso et al., 2021a; Rossanigo et al., 2021). Moreover, the characterization of the magnitude of residual random and systematic errors for each signal allowed the setting of specific reference values to be used to identify poorly performing IMUs that need to be recalibrated or discarded. The characterization of the accelerometers and gyroscopes random errors was performed in accordance with *IEEE 2700–2017 Standard for Sensor Performance Parameter Definitions* (IEEE, 2017). In particular, the accelerometer and gyroscope standard deviation (STD) was computed during a 100 s static acquisition, while the gyroscope bias instability (i.e., slow fluctuations of the measurement offset described as a Gauss-Markov process with zero-mean (Unsal and Demirbas, 2012)) was quantified using the Allan variance during an 8-h static acquisition (El-Sheimy, Hou, and Niu, 2008).

### 2.3 Experimental measurement set-up

**FIGURE 2:**
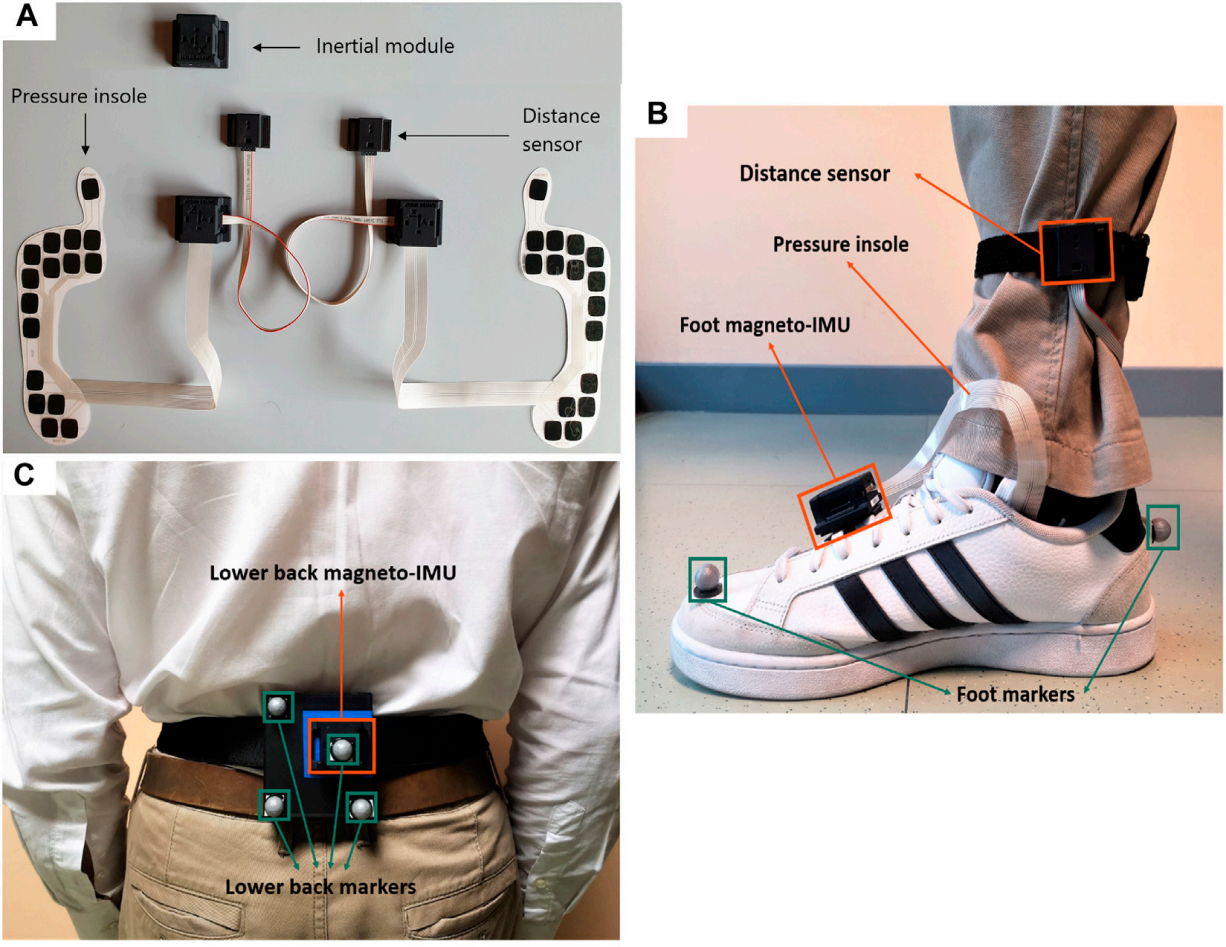
**(A)** Picture of INDIP system hardware. **(B)** Foot positioning, example on right foot (INDIP and stereophotogrammetric system markers). **(C)** Lower back positioning (INDIP and stereophotogrammetric system markers).

A pair of PIs were selected, according to the subject’s foot size, and inserted between the insole and midsole of the shoes. Two magneto-IMUs were positioned over the instep and fixed to the shoelaces using custom-made clips, and a third magneto-IMU was attached to the lower back using an elastic belt with Velcro. To avoid mutual infrared interferences, distance sensors were positioned asymmetrically using Velcro straps (one just above the left ankle and the other about 3 cm higher on the right side), both pointing medially. Both PIs and distance sensors were connected *via* cable to the magneto-IMU of the corresponding foot (Figure 2).

To validate INDIP system, stereophotogrammetric technology was used, as it allows to accurately reconstruct the human movement also under complex motor tasks. In each laboratory, marker trajectories were recorded using the stereophotogrammetric system locally installed (Newcastle: 14-camera Vicon Bonita, Sheffield: 10-camera Vicon MX T160 Vicon Vero, Tel Aviv: 8-camera Vicon T10, Kiel: 12-camera Qualisys Miqus, Stuttgart: 8-camera Vicon T10). A total of eight reflective markers were used: two markers on each foot (heel and toe), and four markers placed on a rigid cluster used as support for the lower back magneto-IMU (Figure 2). For each marker acquisition, a quality control procedure was followed to estimate random and systematic errors of the different stereophotogrammetric systems as described in (Della Croce and Cappozzo, 2000; Scott et al., 2021). The stereophotogrammetric and the INDIP systems, both acquiring at 100 Hz, were synchronized using an additional INDIP magneto-IMU as external trigger, connected to the stereophotogrammetric system *via* USB cable. To this end, the clock of each INDIP magneto-IMU, including the one adopted as external trigger, was set on the same timestamp before each experimental session.

### 2.4 Experimental protocol

The experimental protocol for the validation comprised eight different motor tasks for a total of eleven trials with an increasing level of complexity (Scott et al., 2022). These included simulated daily activities test and seven structured walking tests: Timed-Up and Go, straight walk at comfortable, slow, and fast speed (each repeated twice), L-test, Surface test, and Hallway test. The simulated daily activities test is the most complex and challenging task and was used to capture various daily activities expected in the real-world simulated in a lab environment (i.e., setting the table for dinner, sitting down for a short break, clearing the table etc.). The INDIP was also used during 2.5-h real-world acquisitions to test the usability of the system and the consistency of the extracted DMOs values with those found in literature. In this case, all participants were asked to continue with their daily routine, including some recommended activities such as: walking outside; walking along an inclined path; walking up and down stairs; moving from one room to another, etc. Further details about the experimental protocol can be found in (Mazzà et al., 2021; Scott et al., 2022).

Before any experimental session, each magneto-IMU underwent a preliminary 60 s short static spot-check to compute the gyroscope bias and verify that all the sensors (i.e., accelerometer, gyroscope) were working properly (Picerno, Cereatti, and Cappozzo, 2011). Quality of the PIs signals was checked by applying a direct finger pressure on each sensing unit separately.

### 2.5 Participants

The validation experiments involved two groups of healthy participants—young adults (HYA) and older adults (HOA)—and five cohorts of patients with different diseases that impact mobility (PD, MS, COPD, CHF, PFF), totaling 128 participants (Table 1). All participants provided written informed consent before participating to the study (Ethics approval for the HYA: University of Sheffield Research Ethics Committee, Application number 029143; Ethics approvals for HOA and the cohorts of patients are reported in (Scott et al., 2022).

**TABLE 1:** Cohorts descriptors and clinical parameters of the patient groups.

	Parameters						
Cohort	Participants recruited n)	Gender (M/F)	Age (years, mean ± STD)	Height (m, mean ± STD)	Body mass (kg, mean ± STD)	Walking aid users (n, general use, lab)	Clinical scale
HYA	20	11/9	29.4 ± 9.4	1.74 ± 0.09	70.2 ± 10.1	—	—
HOA	20	11/9	71.7 ± 5.8	1.66 ± 0.10	75.1 ± 11.8	1, 0	—
PD	20	16/4	69.8 ± 7.2	1.73 ± 0.07	78.2 ± 14.4	6, 1	UPDRS III* (mean ± STD, 28.4 ± 13.6)
							H&Y Scale* (I n = 4, II n = 11, III n = 5)
MS	20	11/9	48.7 ± 9.7	1.71 ± 0.13	84.0 ± 22.9	5, 3	EDSS* (mean ± STD, 3.5 ± 1.7)
COPD	17	9/8	69.4 ± 9.1	1.69 ± 0.07	73.7 ± 14.2	1, 0	CAT* (mean ± STD, 16.6 ± 8.9)
							FEV_1*_ (mean ± STD, 1.6 ± 0.6)
CHF	12	8/4	69.1 ± 11.7	1.74 ± 0.10	84.5 ± 16.8	4, 4	KCCQ* (mean ± STD 80.5 ± 20.2)
PFF	19	8/11	80.0 ± 8.5	1.69 ± 0.08	68.4 ± 16.0	13, 6	SPPB* (mean ± STD, 6.2 ± 3.9)

* CAT, COPD assessment test; EDSS, expanded disability status scale; FEV_1_, forced expiratory volume; H&Y, hoehn and yahr scale; KCCQ, kansas city cardiomyopathy questionnaire; MDS-UPDRS, Movement Disorder Society-sponsored Unified Parkinson’s Disease Rating Scale; SPPB, short physical performance battery.

For the lab-based validation, each participant was equipped with the INDIP system and the reflective markers for the stereophotogrammetric system as depicted in Figure 2. For the real-world acquisitions, each participant was equipped with the INDIP system only. In addition, the HYA participating in the real-world experiment (n = 11/20) were asked to fill out a questionnaire regarding the INDIP system usability (Comfort Rating Scale, see Appendix B for more details). Patients were not asked to fill out the questionnaire since this was not the principal aim of the validation study.

### 2.6 Data cleaning, quality check, and processing

Data acquired with the INDIP system, both in laboratory and real-world acquisitions, underwent a quality check procedure and were discarded in case of technical issues associated with 1) partial data loss or synchronization failure due to trigger functioning or to timestamp setting procedure, and/or 2) deteriorated PI data quality. Moreover, stereophotogrammetric recordings were checked in case of gaps longer than 0.5 s due to occlusions (trials with gaps). In particular, trials with gaps were double checked to verify if the identification of the number of strides based on the stereophotogrammetric system was affected by the presence of gaps, in which case they were definitively discarded. Further details are reported in the Results section.

A pre-processing procedure was applied to refine the data synchronization among the INDIP IMUs and the stereophotogrammetric system over each data recording to prevent potential inaccuracy in the sample frequency or differences in clock stability. In particular, the recordings of the three mounted INDIP IMUs (started *via* Bluetooth at the beginning of each trial) were cut and interpolated using a common time vector provided by the external trigger with a synchronization error of ±10 ms (±1 frame).

### 2.7 INDIP algorithms for DMOs estimation

**FIGURE 3:**
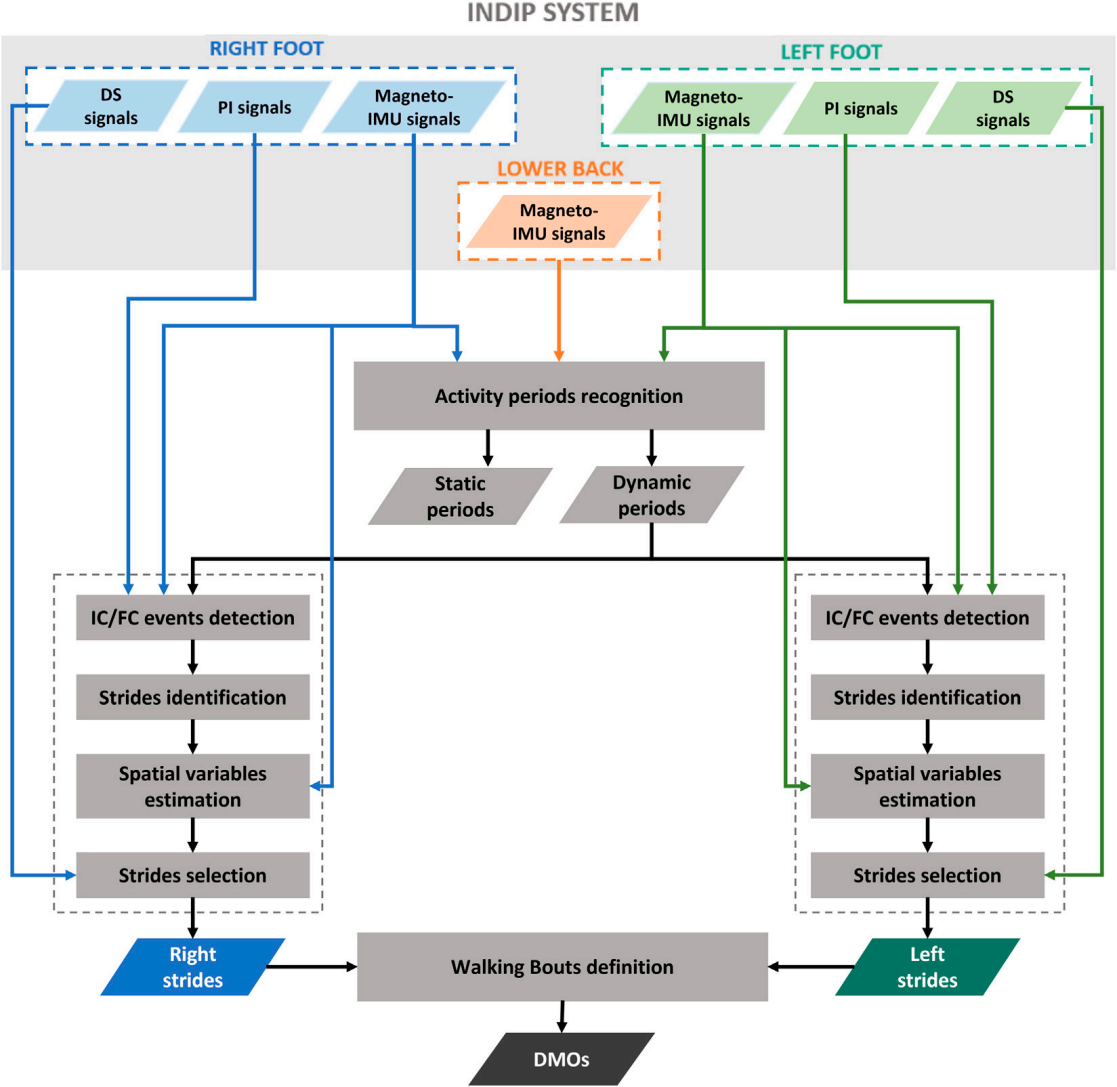
Workflow that shows the principal steps of INDIP algorithm.

The estimation of the relevant spatial-temporal parameters from INDIP data consisted of the following steps, reported as a workflow in Figure 3:

• *Static/dynamic activity periods recognition*: this step was performed to identify dynamic activity intervals potentially including walking. The participant was considered “active” if the standard deviation of the total acceleration of both lower back and at least one foot were above thresholds, empirically chosen (0.7 and 2.1 m/s^2^, respectively) (Lyons et al., 2005; Hickey et al., 2016).
• *Initial contact (IC) and final contact (FC) events detection*: temporal events were detected separately using the information obtained from PIs signals and from magneto-IMUs on the subject’s feet. The PI-method is based on the identification of activation/deactivation clusters of PI sensing elements belonging to the same neighborhood under the hypothesis that, when an IC or FC occurs, the sensing elements referring to the same anatomical region of the foot are activated or deactivated, respectively. A detailed description of the method is provided in (Salis et al., 2021a). The algorithm used to detect gait events from IMU signals is a modified version, adapted for foot mounted IMUs, of that proposed by Trojaniello and colleagues for shank positioning, which exploits invariant kinematic constraints to optimize the IC and FC search (Trojaniello et al., 2014).

Each event obtained from the PIs was associated with the closest event obtained from the feet magneto-IMUs within a tolerance interval of ±0.25 s. An event detected by both magneto-IMUs and PIs, or by the PIs only, was considered as a true event, and the value obtained from the PIs was assigned. The events detected by the feet magneto-IMUs only were included after verifying that the time interval identified between IC and FC corresponded to a stance phase. This was done by applying additional checks based on two detectors typically used for Zero velocity update technique (ZUPT) (Skog et al., 2010): 1) A threshold on the Angular Rate Energy detector signal (0.5 normalized unit). If the values of the angular rate energy were below the threshold for less than 100 ms, the corresponding IC and FC were discarded; 2) A threshold on the Moving Variance detector signal (0.005 normalized unit). If the values of the variance were below the threshold for less than 100 ms, the corresponding IC and FC were discarded.

**FIGURE 4:**
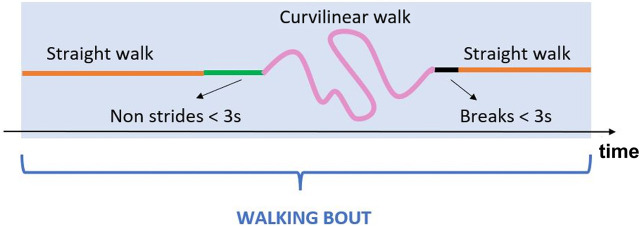
Example of a generic walking bout which includes straight walking, curvilinear walking, non-strides portions and short breaks.

• *Strides identification*: based on the detected temporal gait events, right and left strides were defined as the interval between two consecutive ICs of the same foot.
• *Spatial variables estimation*: stride velocity and displacement were computed from the linear acceleration recorded by feet magneto-IMUs. First, a Madgwick filter was applied to obtain an accurate orientation estimate for each foot magneto-IMU (Madgwick, Harrison, and Vaidyanathan, 2011). This filter was chosen for its simplicity, as it requires the tuning of only one parameter (Caruso et al., 2020; Caruso et al., 2021b), and low computational burden (Caruso et al., 2021a). The parameter value was optimized by minimizing the error obtained on stride length estimates (Rossanigo et al., 2021). The drift associated to the acceleration signal was then reduced taking advantage of the cyclic nature of gait. ZUPT was applied, specifically using the Angular Rate Energy detector to identify the integration intervals, under the hypothesis that foot velocity is negligible during the mid-stance phase (Skog et al., 2010; Skog, Nilsson, and Peter, 2010; Peruzzi, Croce, and Cereatti, 2011). Finally, velocity and displacement were obtained with a direct and reverse integration approach (Zok, Mazzà, and Della Croce, 2004; Trojaniello et al., 2014). In particular, the procedure reported in (Trojaniello et al., 2014), well described in (Zok, Mazzà, and Della Croce, 2004), was adapted to feet positioning, according to what described in (Rossanigo et al., 2021), by exploiting the information obtained from ZUPT to: 1) define each integration instant as the time point in the middle of the corresponding ZUPT interval; 2) correct the velocity estimation in correspondence of the ZUPT intervals before integrating to obtain the displacement.
• *Strides selection*: based on temporal and spatial variables, a selection of right and left strides was performed by applying thresholds on specific stride relevant parameters agreed within the Mobilise-D consortium, including minimum (≥0.2 s) and maximum duration (≤3 s), and minimum length (≥0.15 m). Finally, for each selected stride, measures of the inter-leg distance obtained from the distance sensors were used as a further verification element of the correct stride identification procedure (Bertuletti et al., 2018).
• *Definition of Walking Bouts*: each walking bout was defined starting from the identification of left and right stride sequences separately. Two consecutive ipsilateral strides separated by a time interval lower than 3 s (short break) were considered as belonging to the same stride sequence. Left and right stride sequences were then combined to obtain the walking bouts, according to the matching of the corresponding time sequences. Initial and terminal phases of gait were discarded by removing the first and last stride of each walking bout, since the first and last IC are part of the transition from the previous and following activity, respectively. At this point, eligible walking bouts were selected according to the number of strides they included (minimum two left and two right strides) (Mazzà et al., 2021). An example of walking bout is shown in Figure 4.
• *Calculation of Digital Mobility Outcomes (DMOs)*: a complete set of primary and secondary DMOs were computed for each walking bout (Mazzà et al., 2021). For practical reasons, only a selected DMOs subset is reported:
  o Walking bout duration (WB duration, s), walking bout length (WB length, m), and number of strides, all being informative of the general walking bout characteristics.
  o Cadence (steps/min), being associated with the reliability of ICs identification, and computed as follows:

$$Cadence=\frac{\sum_{j=1}^{\#strides}\left(\frac{60}{Stride\_{Duration}_{j}}\right)}{\#strides}\times 2$$ (1)

o Average stance duration (s) at walking bout level, associated with the reliability of both FCs and ICs identification (swing duration was not reported as it provides similar information).
o Average stride length (m) at walking bout level, being associated with the capability of accurately estimating displacement.
o Walking speed (m/s), informative of the correct estimate of both ICs and displacement, computed as:
$$Walking\;speed=\sum_{j=1}^{\#strides}\frac{Strid{e\_Length}_{j}}{Strid{e\_Duration}_{j}}$$ (2)

### 2.8 Description of the stereophotogrammetry algorithms for DMOs estimation

For the stereophotogrammetry-based method, relevant DMOs were quantified from pelvic and foot marker trajectories according to the method proposed by (Bonci et al., 2022). Briefly, foot trajectories were initially gap-filled only for gaps shorter than 0.5 s and all marker trajectories were filtered using a zero-lag fourth order Butterworth filter (cut-off frequency 7 Hz). As a first approximation, ICs and FCs estimates were chosen in correspondence of the instants of local maxima and minima displacements of the heel and toe markers from the pelvis, respectively. The latter estimates were then refined based on the 3D marker velocities, as detailed in (Bonci et al., 2022). Stride length and speed were measured from the heel marker trajectories between two subsequent ICs of the same foot. Strides and walking bouts were then selected following the same criteria adopted for the INDIP. The quality check procedure followed on the different stereophotogrammetric systems led to systematic errors among the different sites <2.5 mm (Scott et al., 2021).

### 2.9 Statistical analysis

The validation was performed by comparing the results from the INDIP with those provided by the stereophotogrammetric system. The analysis was conducted by aggregating the DMOs values, computed for each walking bout, at cohort level and considering the seven structured tests and the simulated daily activities separately, being the latter the only test that included activities which are different from gait (Scott et al., 2022). It is important to note that, while for the seven structured tests it was expected to detect a single walking bout for each trial, for the simulated daily activities test, a single trial can lead to one or more walking bouts. Only the walking bouts detected by both the systems have been included in the analysis (99% for both structured tests and simulated daily activities).

For example, let us consider a specific cohort composed by *N* subjects, and let suppose that subject *i* performs several trials corresponding to a number of walking bouts equal to $m_{i}$ . The total number of walking bouts for the considered cohort is then

$$M=\sum_{i=1}^{N}m_{i}$$

For each DMO, mean and standard deviation values were computed for both stereophotogrammetric and INDIP systems over the *M* observations equal to the total number of walking bout detected for a given population ( ${\bar{DMO}}_{SP}$ ; ${\bar{DMO}}_{INDIP}$ ).

In addition, for each DMO, errors ( $E_{j}$ *)* and relative errors ( ${E\%}_{j}$ ) for the *j*th walking bout were computed as:

$$E_{j}={DMO}_{INDIP,j}-{DMO}_{SP,j}$$ (3)

$${E\%}_{j}=\frac{{DMO}_{INDIP,j}-{DMO}_{SP,j}}{{DMO}_{SP,j}}\times 100$$ (4)

where ${DMO}_{INDIP,j}$ and ${DMO}_{SP,j}$ are the DMO values obtained from the INDIP system and the stereophotogrammetric system, respectively, for the *j*th walking bout with j = 1: *M*.

As the temporal variables are indirectly derived from stereophotogrammetric system, it is important to note that the values of $E_{j}$ and ${E\%}_{j}$ computed for the temporal DMOs should be regarded as differences between the two systems rather than actual errors.

A normality test (Shapiro-Wilk test) was used to determine, for each cohort and all the relevant DMOs, if errors were normally distributed (Mishra et al., 2019). As the large majority of errors showed a non-normal distribution, median value, median absolute value, and interquartile range value of the errors were computed over the *M* walking bouts detected for the relevant cohort to describe INDIP performance in terms of bias, accuracy, and precision (Walther and Moore, 2005), together with mean value and mean absolute value to allow the comparison with previous studies.

Finally, for each DMO and cohort, the absolute agreement between the two systems was tested using Intraclass Correlation Coefficients (ICC_2,1_: two-way random effects model, absolute-agreement, 95% confidence intervals (Koo and Li, 2016)) computed using SPSS Software, Version 28.0.1.1. Values lower than 0.5, between 0.5 and 0.75, between 0.75 and 0.9, and larger than 0.90 were indicative of poor, moderate, good, and excellent agreement, respectively (Koo and Li, 2016). A statistical power analysis was performed in Stata 16.1 as described in (Scott et al., 2022) to determine the minimum number of walking bouts needed for the validation, according to the desired ICC and the confidence interval values. The values obtained for a confidence interval width of 0.1 were: 401 (ICC ≥ 0.7), 295 (ICC ≥ 0.75), 200 (ICC ≥ 0.8), 119 (ICC ≥ 0.85), 56 (ICC ≥ 0.9) and 16 (ICC ≥ 0.95) walking bouts.

## 3 Results

### 3.1 Sensor noise characterization

**FIGURE 5:**
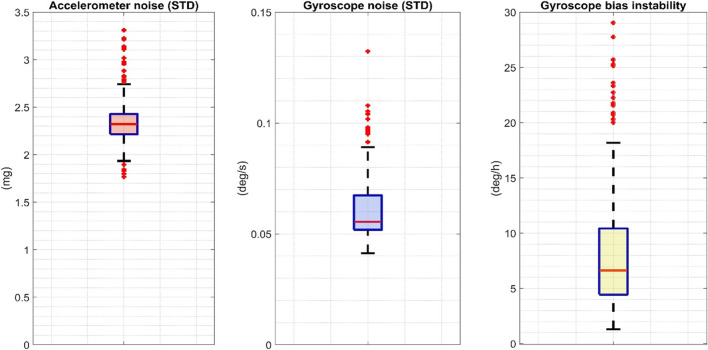
The boxplot distributions of the accelerometer and gyroscope STD and the gyroscope bias computed over 72 INDIP. STD: standard deviation.

The boxplot distributions of the accelerometer STD, gyroscope STD, and gyroscope bias instability computed over the 72 IMUs included in the above-described characterization procedure are shown in Figure 5.

### 3.2 The INDIP performance in laboratory

Across the 128 participants recorded in the laboratory experiments, the majority were able to complete the full protocol (100% for HYA and COPD, 95% for HOA, PD and MS, 92% for CHF and 68% for PFF). Four participants were excluded from the analysis due to technical issues in the acquisitions linked to data loss or synchronization failure (1 MS, 1 CHF, 2 PFF). In addition, data from five participants were discarded due to different technical problems which affected PI data quality (1 PD, 2 MS, 1 CHF, 1 PFF). In the laboratory gait assessment, data obtained from 119/128 participants (completion percentages: 100% HYA and COPD, 95% HOA and PD, 94% MS, 90% CHF, 69% PFF) were included in the analysis.

Among the 119 subjects considered, 44 had at least one trial with a gap in a foot marker trajectory longer than 0.5 s. As a result, 129 trials, out of the total 1,132 trials recorded, required further quality checks prior to inclusion in the analysis. This additional quality check led to 79 of the 129 *trials with gaps* being discarded from the structured tests (27 HOA, 2 PD, 5 MS, 1 COPD, 14 CHF, 30 PFF) and 4 trials from the simulated daily activities (2 HOA, 1 MS, 1 PFF). Overall, 963 walking bouts were analyzed for the structured tasks for a total of 12,749 strides, and 431 walking bouts for the simulated daily activities test including 3,684 strides (Table 2).

**TABLE 2:** Number of Analyzed walking bouts and strides in laboratory (Structured and simulated daily activities tests) and real-world (2.5-h).

Cohort	*Laboratory*				*Real-world*	
	*Structured tests*		SDA* test			
	WBs* (n)	*Strides (n)*	WBs* (n)	*Strides (n)*	WBs* (n)	*Strides (n)*
HYA	189	2072	98	801	470	64,406
HOA	135	1,663	71	483	1,197	43,661
PD	157	2,219	67	593	557	26,812
MS	154	2084	49	494	484	16,493
COPD	135	1826	84	645	1,035	22,127
CHF	73	939	27	235	605	25,283
PFF	120	1946	35	433	531	15,273
*TOTAL*	963	12,749	431	3,684	4,879	213,945

*Abbreviations reported in the table: SDA, simulated daily activities; WBs, walking bouts.

The results obtained from the comparison of INDIP and stereophotogrammetric systems are reported in Table 3 for the structured tests and in Table 4 for the simulated daily activities test. For each cohort and relevant DMO, descriptive statistics (M and STD) of the relevant DMOs values as estimated by INDIP and stereophotogrammetric system values are reported along with the agreement between distributions (ICC values). In addition, the following metrics were reported: mean error, mean absolute error and relative percentage values (*ME*, *MAE, ME%,* and *MAE%*); median error, median absolute error and relative percentage values (*MDE*, *MDAE, MDE%,* and *MDAE%*); interquartile range error and relative percentage value (*IQRE* and *IQRE%*).

**TABLE 3:** Comparison between INDIP and stereophotogrammetric system for the relevant DMOs (structured tests).

DMO	Cohort	M ± STD * (INDIP)	M ± STD * (SP)*	*ME* (*ME%*) *	*MDE* (*MDE%*) *	*IQRE* (*IQRE%*)*	*MAE* (*MAE%*) *	*MDAE* (*MDAE%*) *	ICC_2,1_ *
WB* duration (s)	HYA	7.16 ± 5.40	7.28 ± 5.41	−0.09 (−1.88%)	−0.03 (−0.52%)	0.06 (1.65%)	0.12 (2.39%)	0.03 (0.61%)	0.999
	HOA	7.95 ± 5.63	7.91 ± 5.63	−0.13 (−2.22%)	−0.03 (−0.41%)	0.09 (2.60%)	0.20 (3.09%)	0.04 (0.70%)	0.998
	PD	10.08 ± 7.60	10.29 ± 7.67	−0.07 (−0.91%)	−0.02 (−0.25%)	0.08 (1.42%)	0.26 (3.22%)	0.04 (0.51%)	0.998
	MS	9.80 ± 7.77	10.01 ± 7.83	−0.07 (−0.71%)	−0.01 (−0.11%)	0.08 (1.25%)	0.24 (2.82%)	0.03 (0.46%)	0.998
	COPD	8.93 ± 6.94	8.79 ± 6.88	−0.03 (−0.47%)	−0.01 (−0.10%)	0.06 (1.23%)	0.21 (2.92%)	0.03 (0.63%)	0.998
	CHF	9.16 ± 7.48	9.19 ± 7.35	−0.08 (−1.11%)	−0.03 (−0.42%)	0.07 (1.62%)	0.16 (1.89%)	0.05 (0.75%)	0.999
	PFF	11.58 ± 9.32	11.45 ± 8.96	0.13 (3.29%)	−0.02 (−0.33%)	0.10 (1.54%)	0.39 (4.35%)	0.05 (0.87%)	0.954
WB* length (m)	HYA	7.66 ± 5.56	7.48 ± 5.45	0.02 (0.19%)	0.05 (1.11%)	0.21 (2.78%)	0.22 (3.37%)	0.14 (2.18%)	0.998
	HOA	7.35 ± 5.41	7.11 ± 5.46	−0.06 (−0.44%)	0.03 (0.43%)	0.22 (4.79%)	0.24 (4.16%)	0.11 (2.50%)	0.998
	PD	7.37 ± 5.36	6.99 ± 5.21	0.00 (0.22%)	0.01 (0.20%)	0.29 (5.11%)	0.23 (3.96%)	0.15 (2.44%)	0.998
	MS	7.11 ± 5.28	7.04 ± 5.32	−0.03 (−0.26%)	0.01 (0.10%)	0.26 (4.79%)	0.25 (4.01%)	0.13 (2.46%)	0.997
	COPD	8.03 ± 6.39	7.90 ± 6.29	−0.01 (−0.45%)	0.01 (0.29%)	0.21 (3.61%)	0.21 (3.08%)	0.11 (1.80%)	0.999
	CHF	7.09 ± 5.18	6.70 ± 4.06	−0.04 (−0.30%)	0.02 (0.53%)	0.24 (4.48%)	0.20 (3.02%)	0.11 (2.25%)	0.997
	PFF	6.53 ± 4.74	5.56 ± 3.60	0.01 (1.05%)	0.01 (0.11%)	0.21 (4.17%)	0.29 (5.33%)	0.09 (1.89%)	0.975
Strides number	HYA	10.93 ± 8.97	11.04 ± 8.98	−0.08 (0.91%)	0.00 (0.00%)	0.00 (0.00%)	0.15 (1.64%)	0.00 (0.00%)	0.999
	HOA	12.42 ± 9.92	12.27 ± 9.86	−0.13 (−1.66%)	0.00 (0.00%)	0.00 (0.00%)	0.35 (3.03%)	0.00 (0.00%)	0.997
	PD	14.13 ± 11.68	14.53 ± 11.95	−0.17 (−0.88%)	0.00 (0.00%)	0.00 (0.00%)	0.41 (3.24%)	0.00 (0.00%)	0.998
	MS	13.38 ± 11.16	13.77 ± 11.31	−0.06 (−0.37%)	0.00 (0.00%)	0.00 (0.00%)	0.37 (3.10%)	0.00 (0.00%)	0.998
	COPD	13.53 ± 11.47	13.26 ± 11.37	−0.01 (0.01%)	0.00 (0.00%)	0.00 (0.00%)	0.30 (2.86%)	0.00 (0.00%)	0.998
	CHF	12.74 ± 10.80	12.64 ± 10.55	−0.02 (−0.59%)	0.00 (0.00%)	0.00 (0.00%)	0.25 (1.55%)	0.00 (0.00%)	0.998
	PFF	16.22 ± 13.80	16.15 ± 13.70	0.07 (2.71%)	0.00 (0.00%)	0.00 (0.00%)	1.00 (7.56%)	0.00 (0.00%)	0.970
Cadence (steps/min)	HYA	104.51 ± 18.04	103.45 ± 17.38	1.01 (0.92%)	0.30 (0.32%)	0.90 (0.87%)	1.21 (1.10%)	0.46 (0.46%)	0.990
	HOA	103.04 ± 17.36	102.50 ± 17.11	0.59 (0.58%)	0.23 (0.23%)	0.89 (0.85%)	0.96 (0.95%)	0.41 (0.38%)	0.995
	PD	93.64 ± 16.78	93.27 ± 16.66	0.36 (0.41%)	0.11 (0.10%)	0.76 (0.79%)	0.74 (0.80%)	0.27 (0.28%)	0.995
	MS	93.40 ± 18.48	93.55 ± 18.31	0.48 (0.69%)	0.08 (0.10%)	0.70 (0.78%)	0.89 (1.10%)	0.32 (0.33%)	0.994
	COPD	99.13 ± 18.03	98.96 ± 17.59	0.27 (0.26%)	0.10 (0.10%)	0.59 (0.61%)	0.53 (0.53%)	0.29 (0.29%)	0.998
	CHF	95.60 ± 17.14	94.43 ± 17.04	0.60 (0.61%)	0.22 (0.24%)	1.03 (1.20%)	1.19 (1.24%)	0.61 (0.59%)	0.992
	PFF	96.89 ± 19.53	96.52 ± 19.29	0.36 (0.33%)	0.34 (0.37%)	1.00 (1.02%)	0.91 (0.98%)	0.49 (0.52%)	0.998
Average Stride Length (m)	HYA	1.33 ± 0.17	1.32 ± 0.17	0.00 (0.29%)	0.01 (0.46%)	0.03 (2.19%)	0.03 (1.94%)	0.02 (1.73%)	0.980
	HOA	1.12 ± 0.16	1.13 ± 0.17	0.00 (0.19%)	0.00 (0.19%)	0.03 (2.97%)	0.03 (2.35%)	0.02 (1.44%)	0.968
	PD	1.04 ± 0.23	1.03 ± 0.23	0.01 (0.75%)	0.01 (0.92%)	0.04 (3.91%)	0.03 (2.58%)	0.02 (2.10%)	0.989
	MS	1.06 ± 0.22	1.06 ± 0.22	0.01 (1.09%)	0.01 (0.70%)	0.04 (3.75%)	0.03 (2.98%)	0.02 (1.96%)	0.978
	COPD	1.13 ± 0.15	1.13 ± 0.15	0.00 (0.08%)	0.01 (0.50%)	0.03 (3.09%)	0.02 (2.04%)	0.02 (1.49%)	0.986
	CHF	1.12 ± 0.26	1.12 ± 0.25	0.00 (0.02%)	0.00 (−0.04%)	0.04 (4.10%)	0.03 (2.46%)	0.02 (2.15%)	0.990
	PFF	0.88 ± 0.32	0.87 ± 0.32	0.00 (1.22%)	0.00 (0.27%)	0.03 (3.28%)	0.02 (3.85%)	0.01 (1.67%)	0.993
Walking Speed (m/s)	HYA	1.17 ± 0.30	1.15 ± 0.30	0.01 (1.22%)	0.01 (1.24%)	0.02 (2.07%)	0.02 (2.23%)	0.02 (1.82%)	0.993
	HOA	0.97 ± 0.25	0.97 ± 0.25	0.01 (0.95%)	0.01 (0.66%)	0.03 (3.27%)	0.02 (2.34%)	0.01 (1.62%)	0.989
	PD	0.82 ± 0.30	0.81 ± 0.29	0.01 (1.16%)	0.01 (1.19%)	0.03 (3.83%)	0.02 (2.67%)	0.02 (2.16%)	0.996
	MS	0.84 ± 0.29	0.84 ± 0.28	0.00 (0.31%)	0.01 (0.94%)	0.03 (3.47%)	0.02 (2.91%)	0.02 (2.07%)	0.994
	COPD	0.94 ± 0.25	0.94 ± 0.26	0.00 (0.30%)	0.01 (0.78%)	0.03 (2.87%)	0.02 (2.09%)	0.01 (1.64%)	0.992
	CHF	0.92 ± 0.34	0.90 ± 0.33	0.01 (0.67%)	0.00 (0.37%)	0.04 (4.18%)	0.02 (2.44%)	0.02 (2.01%)	0.996
	PFF	0.73 ± 0.35	0.72 ± 0.35	0.01 (1.57%)	0.00 (0.59%)	0.02 (3.39%)	0.02 (3.71%)	0.01 (1.74%)	0.996
Average Stance Duration (s)	HYA	0.78 ± 0.16	0.78 ± 0.17	0.00 (−0.02%)	0.00 (0.12%)	0.02 (2.38%)	0.02 (2.29%)	0.02 (2.02%)	0.990
	HOA	0.81 ± 0.16	0.80 ± 0.17	0.01 (1.30%)	0.01 (0.79%)	0.03 (3.88%)	0.02 (3.16%)	0.01 (2.05%)	0.978
	PD	0.89 ± 0.20	0.90 ± 0.21	−0.01 (−0.55%)	0.00 (−0.24%)	0.04 (4.15%)	0.03 (2.79%)	0.02 (2.28%)	0.984
	MS	0.91 ± 0.26	0.92 ± 0.27	−0.01 (1.64%)	−0.01 (−0.92%)	0.04 (4.16%)	0.03 (3.30%)	0.02 (2.20%)	0.975
	COPD	0.83 ± 0.16	0.84 ± 0.16	−0.01 (−1.00%)	−0.01 (−0.63%)	0.03 (4.17%)	0.02 (2.36%)	0.01 (1.84%)	0.986
	CHF	0.87 ± 0.20	0.89 ± 0.20	−0.01 (−1.76%)	−0.01 (−0.89%)	0.04 (4.24%)	0.03 (3.32%)	0.02 (2.21%)	0.976
	PFF	0.89 ± 0.26	0.90 ± 0.27	−0.01 (−1.48%)	−0.01 (−0.95%)	0.05 (5.81%)	0.04 (4.06%)	0.03 (2.93%)	0.975

*Abbreviations reported in the table: M ± STD: mean ± standard deviation; *ME (ME%)*: mean error (mean percentage error); *MDE (MDE%)*, median error (median percentage error); *IQRE (IQRE%)*, interquartile range error (interquartile range percentage error); *MAE (MAE%)*, mean absolute error (mean absolute percentage error); *MDAE (MDAE%)*, median absolute error (median absolute percentage error); *ICC*_*2,1*_, intraclass correlation coefficient, SP, stereophotogrammetric; WB, walking bout.

**TABLE 4:** Comparison between INDIP and stereophotogrammetric system for the relevant DMOs (simulated daily activities test).

DMO	Cohort	M ± STD * (INDIP)	M ± STD * (SP)	*ME* (*ME%*) *	*MDE* (*MDE%*) *	*IQRE* (*IQRE%*) *	*MAE* (*MAE%*) *	*MDAE* (*MDAE%*) *	ICC_2,1_ *
WB* duration (s)	HYA	7.83 ± 2.72	7.93 ± 2.71	−0.09 (−1.88%)	−0.03 (−0.52%)	0.72 (9.76%)	0.52 (6.49%)	0.22 (3.28%)	0.980
	HOA	6.00 ± 1.36	5.91 ± 1.36	−0.13 (−2.22%)	−0.03 (−0.41%)	0.78 (15.92%)	0.80 (14.73%)	0.41 (6.51%)	0.861
	PD	8.34 ± 3.46	8.68 ± 3.68	−0.07 (−0.91%)	−0.02 (−0.25%)	1.08 (13.23%)	0.89 (10.41%)	0.70 (7.54%)	0.963
	MS	9.20 ± 2.59	9.26 ± 2.68	−0.07 (−0.71%)	−0.01 (−0.11%)	1.39 (18.00%)	1.02 (12.56%)	0.69 (9.20%)	0.943
	COPD	6.94 ± 1.29	6.73 ± 1.22	−0.03 (−0.47%)	−0.01 (−0.10%)	1.18 (14.87%)	0.87 (13.24%)	0.08 (1.93%)	0.894
	CHF	10.62 ± 3.95	11.10 ± 4.33	−0.08 (−1.11%)	−0.03 (−0.42%)	0.13 (2.06%)	0.80 (6.64%)	0.06 (1.03%)	0.952
	PFF	10.60 ± 2.78	10.05 ± 2.73	0.13 (3.29%)	−0.02 (−0.33%)	1.44 (13.87%)	1.48 (20.03%)	0.66 (6.56%)	0.762
WB* length (m/s)	HYA	4.64 ± 1.35	4.30 ± 1.55	0.02 (0.19%)	0.05 (1.11%)	0.32 (8.49%)	0.26 (7.84%)	0.16 (4.21%)	0.970
	HOA	3.03 ± 0.54	3.18 ± 0.65	−0.06 (−0.44%)	0.03 (0.43%)	0.41 (14.38%)	0.33 (14.73%)	0.20 (5.94%)	0.938
	PD	3.59 ± 1.29	4.07 ± 2.03	0.00 (0.22%)	0.01 (0.20%)	0.56 (16.13%)	0.34 (10.46%)	0.24 (7.77%)	0.974
	MS	4.76 ± 1.53	4.03 ± 0.95	−0.03 (−0.26%)	0.01 (0.10%)	0.43 (17.84%)	0.30 (10.94%)	0.28 (7.41%)	0.981
	COPD	3.48 ± 1.14	3.39 ± 1.21	−0.01 (−0.45%)	0.01 (0.29%)	0.27 (8.42%)	0.19 (9.36%)	0.09 (3.75%)	0.980
	CHF	4.52 ± 1.64	4.22 ± 1.02	−0.04 (−0.30%)	0.02 (0.53%)	0.17 (6.79%)	0.19 (5.43%)	0.09 (2.65%)	0.981
	PFF	4.09 ± 1.78	3.63 ± 1.05	0.01 (1.05%)	0.01 (0.11%)	0.26 (8.82%)	0.34 (12.01%)	0.25 (6.82%)	0.944
Strides number	HYA	8.91 ± 3.25	9.09 ± 3.27	−0.08 (0.91%)	0.00 (0.00%)	1.00 (14.83%)	0.79 (8.62%)	1.00 (7.14%)	0.965
	HOA	7.12 ± 1.75	6.95 ± 1.89	−0.13 (−1.66%)	0.00 (0.00%)	1.00 (20.00%)	0.98 (15.20%)	1.00 (12.50%)	0.888
	PD	8.89 ± 3.78	9.08 ± 4.06	−0.17 (−0.88%)	0.00 (0.00%)	1.00 (13.29%)	0.97 (11.70%)	1.00 (9.76%)	0.962
	MS	11.14 ± 3.67	11.34 ± 3.57	−0.06 (−0.37%)	0.00 (0.00%)	2.00 (20.20%)	1.27 (11.80%)	1.00 (11.11%)	0.961
	COPD	7.87 ± 2.10	7.57 ± 2.19	−0.01 (0.01%)	0.00 (0.00%)	2.00 (20.00%)	1.07 (15.72%)	1.00 (9.09%)	0.912
	CHF	10.81 ± 3.80	11.32 ± 4.12	−0.02 (−0.59%)	0.00 (0.00%)	1.00 (11.11%)	0.73 (6.42%)	0.00 (0.00%)	0.968
	PFF	12.53 ± 4.02	11.91 ± 3.02	0.07 (2.71%)	0.00 (0.00%)	3.50 (35.00%)	2.34 (23.68%)	2.00 (17.64%)	0.815
Cadence (steps/min)	HYA	86.50 ± 9.94	85.27 ± 8.97	1.01 (0.92%)	0.30 (0.32%)	3.09 (3.74%)	2.72 (3.45%)	1.07 (1.39%)	0.929
	HOA	93.99 ± 8.97	91.64 ± 7.58	0.59 (0.58%)	0.23 (0.23%)	5.04 (5.92%)	4.36 (4.97%)	2.78 (2.62%)	0.867
	PD	83.40 ± 8.09	82.41 ± 10.18	0.36 (0.41%)	0.11 (0.10%)	3.35 (4.33%)	4.02 (4.61%)	2.14 (2.72%)	0.871
	MS	89.26 ± 8.38	87.63 ± 8.79	0.48 (0.69%)	0.08 (0.10%)	4.43 (5.01%)	3.14 (3.77%)	2.19 (2.45%)	0.902
	COPD	89.07 ± 9.54	86.32 ± 9.55	0.27 (0.26%)	0.10 (0.10%)	5.31 (6.29%)	4.87 (6.28%)	2.15 (2.42%)	0.733
	CHF	85.83 ± 11.23	82.27 ± 6.02	0.60 (0.61%)	0.22 (0.24%)	2.59 (2.74%)	3.26 (4.26%)	1.13 (1.37%)	0.740
	PFF	87.81 ± 10.31	88.50 ± 10.41	0.36 (0.33%)	0.34 (0.37%)	4.20 (4.32%)	3.63 (4.17%)	1.83 (2.39%)	0.918
Average Stride Length (m)	HYA	0.99 ± 0.14	0.97 ± 0.14	0.00 (0.29%)	0.01 (0.46%)	0.06 (7.25%)	0.05 (7.20%)	0.04 (3.77%)	0.965
	HOA	0.83 ± 0.17	0.80 ± 0.11	0.00 (0.19%)	0.00 (0.19%)	0.09 (12.34%)	0.06 (9.96%)	0.04 (5.50%)	0.939
	PD	0.74 ± 0.13	0.72 ± 0.16	0.01 (0.75%)	0.01 (0.92%)	0.05 (8.07%)	0.05 (8.77%)	0.04 (5.71%)	0.963
	MS	0.81 ± 0.16	0.79 ± 0.13	0.01 (1.09%)	0.01 (0.70%)	0.09 (11.83%)	0.06 (7.88%)	0.04 (6.01%)	0.934
	COPD	0.82 ± 0.11	0.82 ± 0.12	0.00 (0.08%)	0.01 (0.50%)	0.05 (8.82%)	0.04 (6.99%)	0.03 (3.73%)	0.981
	CHF	0.80 ± 0.14	0.81 ± 0.13	0.00 (0.02%)	0.00 (−0.04%)	0.04 (6.51%)	0.05 (7.17%)	0.02 (2.21%)	0.927
	PFF	0.63 ± 0.12	0.62 ± 0.11	0.00 (1.22%)	0.00 (0.27%)	0.09 (16.07%)	0.05 (8.95%)	0.05 (8.89%)	0.939
Walking Speed (m/s)	HYA	0.73 ± 0.16	0.71 ± 0.16	0.01 (1.22%)	0.01 (1.24%)	0.05 (10.86%)	0.04 (8.24%)	0.03 (4.94%)	0.978
	HOA	0.66 ± 0.18	0.63 ± 0.11	0.01 (0.95%)	0.01 (0.66%)	0.06 (13.32%)	0.05 (11.21%)	0.03 (6.82%)	0.942
	PD	0.52 ± 0.13	0.51 ± 0.11	0.01 (1.16%)	0.01 (1.19%)	0.03 (8.38%)	0.03 (8.31%)	0.02 (5.20%)	0.975
	MS	0.61 ± 0.15	0.58 ± 0.13	0.00 (0.31%)	0.01 (0.94%)	0.08 (12.70%)	0.05 (9.14%)	0.03 (7.03%)	0.944
	COPD	0.61 ± 0.10	0.60 ± 0.10	0.00 (0.30%)	0.01 (0.78%)	0.04 (8.91%)	0.03 (8.23%)	0.02 (3.33%)	0.983
	CHF	0.58 ± 0.08	0.57 ± 0.10	0.01 (0.67%)	0.00 (0.37%)	0.05 (10.08%)	0.03 (6.60%)	0.02 (3.20%)	0.973
	PFF	0.46 ± 0.08	0.46 ± 0.07	0.01 (1.57%)	0.00 (0.59%)	0.06 (13.75%)	0.04 (8.91%)	0.03 (7.10%)	0.939
Average Stance Duration (s)	HYA	1.04 ± 0.11	1.08 ± 0.14	0.00 (−0.02%)	0.00 (0.12%)	0.09 (6.94%)	0.07 (6.61%)	0.05 (5.10%)	0.860
	HOA	0.97 ± 0.12	0.98 ± 0.12	0.01 (1.30%)	0.01 (0.79%)	0.12 (12.13%)	0.10 (9.72%)	0.06 (6.06%)	0.737
	PD	1.07 ± 0.12	1.09 ± 0.12	−0.01 (−0.55%)	0.00 (−0.24%)	0.11 (9.76%)	0.07 (5.96%)	0.05 (4.18%)	0.911
	MS	1.00 ± 0.14	1.05 ± 0.15	−0.01 (1.64%)	−0.01 (−0.92%)	0.11 (10.62%)	0.08 (7.81%)	0.06 (5.48%)	0.716
	COPD	1.03 ± 0.09	1.07 ± 0.09	−0.01 (−1.00%)	−0.01 (−0.63%)	0.07 (6.39%)	0.07 (6.44%)	0.03 (3.82%)	0.828
	CHF	1.10 ± 0.22	1.21 ± 0.35	−0.01 (−1.76%)	−0.01 (−0.89%)	0.06 (6.08%)	0.11 (7.65%)	0.03 (3.15%)	0.690
	PFF	1.01 ± 0.14	1.03 ± 0.15	−0.01 (−1.48%)	−0.01 (−0.95%)	0.11 (11.79%)	0.08 (8.13%)	0.05 (5.75%)	0.854

*Abbreviations reported in the table: M ± STD: mean ± standard deviation; *ME (ME%)*, mean error (mean percentage error); *MDE (MDE%)*, median error (median percentage error); *IQRE (IQRE%)*, interquartile range error (interquartile range percentage error); *MAE (MAE%)*, mean absolute error (mean absolute percentage error); *MDAE (MDAE%)*, median absolute error (median absolute percentage error); *ICC*_*2,1*_, intraclass correlation coefficient; SP, stereophotogrammetric; WB, walking bout.

#### 3.2.1 Results from the structured tests

An excellent absolute agreement (ICC >0.95) was observed for the structured tests results in all cohorts and DMOs (Table 3). Moreover, the sample size resulted to be adequate in all cases. Considering the results obtained from all the cohorts, the structured tests showed, for all the DMOs, an *MDE%* between −1.0% and 1.3% and *ME%* between −2.22% and 3.29%. The absolute errors were very limited for all cohorts and DMOs, with *MDAE* values consistently lower than *MAE* values (*MDAE*: WB duration ≤0.05 s, WB length ≤0.14 m, average stance duration ≤0.03 s, average stride length ≤0.02 m, walking speed ≤0.02 m/s and cadence ≤0.61 steps/min; *MAE*: WB duration ≤0.39 s, WB length ≤0.29 m, average stance duration ≤0.04 s, average stride length ≤0.03 m, walking speed ≤0.02 m/s and cadence ≤1.21 steps/min).

In terms of percentage errors, we found *MDAE%* values <1% for WB duration and cadence, ≤2.1% for average stride length and walking speed, <3% for WB length and average stance duration. Stride number *MDAE%* observed are equal to zero in every case, as proof of a correct walking bout detection. The *MAE%* values were <5% across DMOs and cohorts except for slightly larger errors on stride numbers in PFF cohorts (7.6%, slowest cohort).

#### 3.2.2 Results from the simulated daily activities test

Regarding this test, the same metrics are extracted for all the cohorts and DMOs (Table 4). The absolute agreement was excellent (ICC >0.90) in all the cohorts for WB length, average stride length, and walking speed, while it was between excellent and good for the remaining DMOs, except for few cases in which a moderate reliability, with ICC values ≥0.69, was observed (COPD, CHF for cadence and HOA, MS, and CHF for average stance duration, respectively). The sample size was adequate in all cohorts for WB length and walking speed, while analyses for some DMOs-cohorts combinations were under-powered (HOA and PFF for WB duration; PFF for stride number; HOA, PD, COPD, and CHF for cadence; HOA, MS, COPD, CHF, and PFF for average stance duration).

Strides number shows a zero bias for all cohorts (*ME* between −0.45 and 0.34), while the *MDAE* are between 0 (CHF, *MAE* 0.73) and 2 (PFF, *MAE* 2.34) across cohorts, with *MDAE%* ranging from 0% (CHF, *MAE%* 6.42%) to 17.64% (PFF, *MAE%* 23.68%). Due to the differences in strides number, also the *MDAE* and *MAE* obtained for the other DMOs were in general moderately higher with respect to those obtained for the structured tests. For instance, *MDAE* for walking speed ranged between 0.02 m/s and 0.03 m/s (*MAE* between 0.03 m/s and 0.05 m/s), while *MDAE%* ranged between 3.2% and 7.1% (*MAE* between 6.6% and 11.21%).

### 3.3 The INDIP real-world outcomes

The same participants were also involved in a 2.5-h unsupervised recording (except for the HYA for which we have a subset of 11/20 subjects) for a total of 119 participants. The duration of the acquisition reached the expected value in most of the cases (89%, the remaining 11% had a recording duration between 27 and 123 min). Five participants were excluded due to technical issues in the acquisitions (1 PD, 1 MS, 3 PFF), while 18 participants were discarded due to different technical problems which affected PI data quality during the recordings (3 HOA, 4 PD, 5 MS, 1 CHF, 5 PFF) (see Paragraph 2.6). Results on the real-world acquisitions are hence computed on 96/119 participants (81%). For the real-world experiments, 4,879 walking bouts were analyzed including 213,945 strides (Table 2).

**FIGURE 6:**
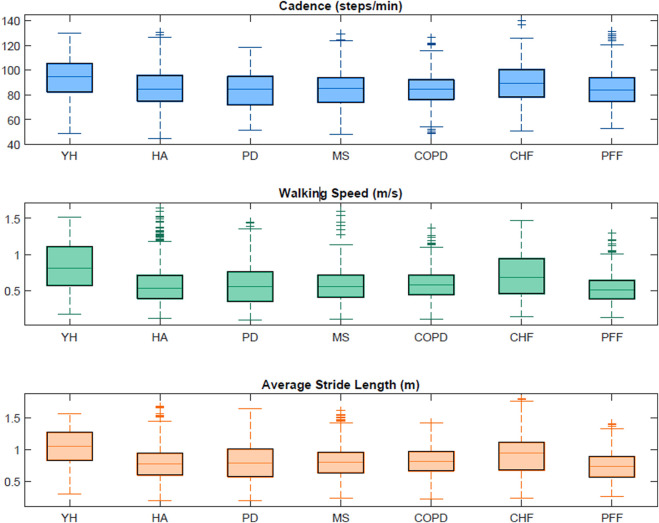
Boxplots obtained from the INDIP system for cadence, average stride length and walking speed for each cohort in the 2.5-h acquisitions.

Table 5 includes the characteristics (min, max and interquartile range values) of the walking bouts detected with the INDIP system in terms of WB duration, WB length and strides number for each cohort. Figure 6 shows the boxplots obtained for a subset of DMOs (cadence, average stride length and walking speed) for each group of participants. Results from the usability questionnaires filled by the 11 HYA are reported in Appendix B.

**TABLE 5:** INDIP Outcomes for duration, total length and strides number (2.5-h real world experiments).

	Cohort	Min-max values*	IQR* value
WB* duration (s)	HY	2.79–1,442.70	46.72
	HA	2.31–1,493.59	12.52
	PD	2.74–1741.60	14.90
	MS	2.82–814.49	15.55
	COPD	2.44–638.52	11.60
	CHF	2.36–1,090.32	13.76
	PFF	2.59–381.21	16.77
WB* length (m)	HY	0.75–2,105.02	42.47
	HA	0.51–1913.74	7.28
	PD	0.49–2,430.50	9.32
	MS	0.62–1,225.93	8.55
	COPD	0.60–699.69	6.28
	CHF	0.59–1,586.70	10.74
	PFF	0.76–425.96	7.71
Strides number	HY	4–2,779	74
	HA	4–2,450	17
	PD	4–3,101	20
	MS	4–1,634	20
	COPD	4–1,081	15
	CHF	4–1766	20
	PFF	4–643	22

*Abbreviations used in the table: IQR, interquartile range; Min, minimum; Max, maximum; WB, walking bout.

## 4 Discussion

In this study, we presented and validated the INDIP, a multi-sensor wearable system specifically conceived for gait assessment under ecologically valid conditions. The system was deployed within the Mobilise-D project (Mobilise-D 2019) for assessing the technical validity of the DMOs estimated based on a single-device attached to the lower trunk for long-term daily-life mobility assessment (Mazzà et al., 2021; Micó-amigo et al., 2022; Scott et al., 2022).

### 4.1 INDIP hardware and algorithms

To ensure transparency, reproducibility and replicability, a thorough description of INDIP system hardware have been provided. Moreover, each of the state-of-the-art algorithms included in the INDIP computational pipeline has been previously described, validated under standard and controlled conditions (Trojaniello et al., 2014; Bertoli et al., 2018; Bertuletti et al., 2019), and specifically optimized for gait assessment. It is important to highlight that, to compute the DMOs according to the definition and the minimum requirements for strides and walking bouts (as agreed within the Mobilise-D consortium (Kluge et al., 2021)), it is necessary to perform a stride-by stride resolution gait analysis, independently of the DMOs aggregation level (e.g., across walking bout, across subjects, across cohort). Temporal gait events were directly measured from the foot-ground contacts detected using 16 force-resistive sensors integrated in the PIs, applying a clustering approach for increasing robustness to noise (Salis et al., 2021a). Similarly, spatial parameters were determined based on the double integration of accelerometric signals recorded by the IMUs attached to the feet, which may benefit from gravity removal and zero-velocity update techniques for noise reduction during walking (Sabatini, 2005; Skog et al., 2010; Rebula et al., 2013; Trojaniello et al., 2014).

### 4.2 INDIP calibration refinement and noise description

The quality and uniformity of the sensor data collected during the experiments were rigorously verified. In fact, the performances of low-cost miniaturized IMUs, commonly employed in human movement monitoring, aren’t as stable as those of IMUs used in navigation applications (Nez et al., 2016). For this reason, it is good practice—when possible—to perform appropriate quality checks, and to eventually refine calibration parameters based on in-field procedures proposed in the literature (Stančin and Tomažič, 2014). In this validation study, sensor characterization and recalibration were performed on all the 72 IMUs used by the five laboratories to verify that each sensor had similar metrological performance and thus facilitating the comparison of the results obtained for the different centers. Furthermore, the description of the noise statistics for both the accelerometers and gyroscopes deployed enabled the setting of reference values for sensor stochastic noise, and the elimination of those sensors which did not satisfy metrological requirements (two IMUs with STD values exceeding by 15% the STD maximum values found for the accelerometers and the gyroscopes distributions–3.31 mg and 0.13 dps, respectively).

### 4.3 INDIP performance validation

A key aspect of this study concerns the efforts devoted to the assessment of the INDIP system performance (Mazzà et al., 2021). In principle, when establishing a new reference method, attention should be paid in validating the estimated DMOs under conditions similar to those of its intended use, that in this context are represented by real-world mobility. However, in practice, this is often not possible due to the lack of well-established valid gold standard solutions for the entire set of gait metrics of interest (Del Din et al., 2016). To overcome this paradox, we tested the INDIP system through an experimental protocol specifically designed and validated (Scott et al., 2022) for simulating several real-world walking conditions in terms of: 1). Complexity and heterogeneity of the motor tests recorded including not only straight walking but also turnings, obstacles, different surfaces, standing and sitting on a chair and intermittent gait due to interaction with objects of the typical home daily life; 2). Types of target populations analyzed (seven different cohorts including normal gait in young and older adults, neurological disorders, orthopedic pathologies, and cardio-respiratory disorders); 3). Broad range of walking speeds, from 0.46 m/s (PFF, simulated daily activities test) to 1.15 m/s (HYA, structured tests) on average; 4). Technical reproducibility (multi-centric data collection carried on five different gait analysis laboratories).

In general, the INDIP system showed very good performance, similar across motor tests and cohorts, supporting the robustness of algorithm’s estimate for a large variety of gait patterns. In particular, the results of the structured motor tests showed excellent concurrent validity between the stereophotogrammetry and INDIP estimates, with ICC values ranging between 0.95 and 0.99 across cohorts and DMOs. Similarly, the accuracy was very high for all the DMOs-cohorts combinations, with *MDAE%* less than 2.93% (Table 3). Precision as represented by interquartile range values was very good for all DMOs and cohorts (<5.2%) with the largest dispersion observed for average stance duration in PFF (5.81%), which is also the cohort with the most frequent use of walking aids (in general, 13/19 PFF patients recruited were walking aid users, Table 1).

As expected, slightly larger errors were observed for the simulated daily activities test, which is characterized by multiple shorter walking bouts separated by motor activities other than walking (i.e., setting the table, moving chair and other objects, etc.). On average, the WB length for this test (3–4 m) was half that observed for the structured tests, resulting in an inevitable increase in DMOs relative errors. For instance, a difference of a single stride between the stereophotogrammetric system and INDIP system led to a relative error from 7.14% to 12.50% depending on the specific cohort analyzed. In general, concurrent validity was excellent for all cohorts for both average stride length and walking speed (ICC >0.94) whereas a larger variability was observed for average stance duration and cadence (ICC values between 0.71 and 0.93). Accuracy level was also good with *MDAE* of 1-2 strides for stride number, smaller than 0.05 m for the average stride length (*MDAE%* ≤ 8.89%), and smaller than 0.03 m/s for walking speed (*MDAE%* ≤ 7.10%) across cohorts.

As the DMOs error distributions were negatively skewed, mean errors were higher with respect to median errors. In fact, the last ones are less sensitive to outliers due to the asymmetry that characterizes error distributions and the data cleaning procedure applied. Interestingly, the INDIP system showed similar performance across tests and cohorts. These findings support the robustness of the algorithm’s estimate for a large variety of different gait patterns. It should be also highlighted that the INDIP system performance was assessed on relatively short walking bouts (length <8 m; number of strides <16.5) which represent critical and challenging experimental conditions compared to motor tests including longer walking bout characterized by more regular and predictable gait patterns (Micó-amigo et al., 2022). This is the worst-case situation, thereby yielding the most conservative estimates.

### 4.4 Comparison with the literature

The choice of reporting both mean and median errors enabled a direct comparison of the results with studies based on different metrics (Trojaniello et al., 2014; Bertoli et al., 2018; Bonci et al., 2022; Micó-Amigo et al., 2022). It is interesting noting that the errors associated with the spatial-temporal parameters estimated by the INDIP were in general larger than those reported by (Trojaniello et al., 2014) and (Bertoli et al., 2018), from which INDIP IMU-based algorithms were derived and refined. Although a direct errors comparison is not possible as errors were computed at different aggregation levels (stride-level versus walking bout level), it is possible to observe that, in Bertoli et al. (Bertoli et al., 2018), stride length mean absolute errors were on average 2% (about 25 mm) for PD patients, compared to errors of 30 mm (structured tests) and 50 mm (simulated daily activities test) found with the INDIP system for the same cohort. Such differences may be explained considering that the original methods (Trojaniello et al., 2014; Bertoli et al., 2018) were validated on gait data recorded for 1 min while the subject was walking on a 12-m-long straight walkway, without including much more complex and challenging motor tests as in the present study. These observations further support the importance of testing the proposed methods under conditions like those usually encountered in real world scenarios (intermittent walking including turning, short walking bouts, breaks and higher gait variability).

In the last decades, several methods based on wearable sensors for mobility assessment (Iosa et al., 2016) have been developed, with a particular attention to feet/shanks IMUs approaches. However, in most of the studies, validation was limited to straight walking, normal gait, or to the evaluation of temporal parameters only. For example, Gastaldi and colleagues (Gastaldi et al., 2015) compared the results obtained from two IMUs with those of a footswitch-based system (STEP 32 footswitches); data were collected on one healthy subject while walking on a 12 m straight path for three times, obtaining relative errors below 5% for cadence computed at trial level. Also Zhou and colleagues (Zhou et al., 2020) tested an algorithm based on two feet mounted IMUs (Physiolog→5 IMUs, Gait Up) against an OptoGait system, using straight walk data collected on five young healthy participants. The stride-by-stride comparison led to root mean square errors of 0.05 m (3%) for stride length. The results obtained with the INDIP system under similar conditions (healthy participants for the structured tests), showed smaller errors both for cadence (MAE% about 1%) and average stride length (MAE 0.03 m). Jakob and colleagues (Jakob et al., 2021) validated a wearable system (Portabiles-HCT GaitLab-System, including two IMUs positioned inside the shoes) on 33 PD patients during straight walk, using the stereophotogrammetry as reference. The method performance was evaluated in terms of ICC values and results were excellent (0.986 for walking speed and 0.985 for stride length) but lower than found with the INDIP system (ICC of 0.996 for walking speed and 0.989 for average stride length in PD patients during structured tests).

Recently, Romijnders and colleagues (Romijnders et al., 2021) stressed the importance of assessing the performance of methods for daily-life use during curved walking and dual-task conditions. With this purpose, they proposed and validated a shank IMU-based algorithm for gait events detection on HOA, PD patients and stroke patients walking in three conditions (straight walk, slalom walk, and dual task walk along an elliptical path). Very good performances were found in terms of recall, precisions against the stereophotogrammetric system (recall between 85% and 100%, precision 95%–100% for HOA and PD). The INDIP system showed similar or better performance, in terms of accuracy based on the number of detected strides (97% for HOA and 98% for PD), across more complex motor tasks.

More recently, it has become evident that there is a need of extending the validation during real-world conditions, comparing IMU-based methods against pressure insoles for the estimation of temporal parameters. For example, Storm and colleagues (Storm, Buckley, and Mazzà, 2016) validated two algorithms, one based on two shank IMUs and the other on one waist IMU, using pressure insoles as reference. Data were collected on ten healthy participants, both indoor and outdoor, while performing straight walking and curvilinear walking, for a total of five different tasks. Among the gait parameters presented, also stance duration was computed, obtaining an average absolute error around 0.04 s for the shank method and around 0.03 s for the waist method across all the tasks. Another relevant work is that proposed by Roth and colleagues (Roth et al., 2021b), in which they validated a pipeline based on foot mounted IMUs against force sensitive resistor pressure insoles. Their performance was evaluated using data collected on 20 healthy participants in supervised real-world conditions (level walking, stairs ascending and stairs descending at normal, slow and fast speed). The authors reported mean absolute error on stance duration about 0.02 s on level walking, 0.03 s ascending, 0.02 s descending, comparable with those obtained from the INDIP based method for the average stance duration in the structured tests (MAE 0.02 s for both HYA and HOA).

Some studies have proposed to use a multi-technology approach for gait analysis (Schepers et al., 2009; Van Meulen et al., 2016; Li et al., 2018; Refai et al., 2018; Tang et al., 2019; Duong et al., 2022), but very few studies characterized the performance of those systems in estimating DMOs against a ground truth reference. An interesting but preliminary study was presented by Li and colleagues (Li et al., 2018), who developed a multi-sensor system including three force sensors (positioned at the heel, arch and forefoot to detect IC and FC), an IMU and four range sensors for each foot. The study involved four healthy male participants and the stereophotogrammetric system was used as reference, obtaining average relative errors - computed among all subjects and trials—of 9.34% for stride length and 5.90% for stride velocity on straight walks (against a MAE% of 2.23% for walking speed and 1.94% for average stride length in HYA from INDIP system). A multi-sensor system with a sensor configuration similar to the INDIP has been recently proposed by Duong and colleagues (*SportSole II)* (Duong et al., 2022). It includes two instrumented insoles, with eight force sensitive resistor elements, each connected to an IMU attached to the shoe. Data were collected on eleven HYA while performing a series of different activities (including tasks with straight walk, curves and stairs). However, the system performance was validated only for selected gait portions (the subject walking on the instrumented walkway during straight or curvilinear portions), and on normal gait. Data were processed using a support vector regression (SVR) based algorithm, obtaining a good performance (*MAE%* structured session: 2.97% for stride length and 3.16% for stride velocity; *MAE%* unstructured session: 3.55% for stride length and 3.59% for stride velocity), but lower than that obtained with the INDIP for the structured tests (*MAE%* 1.94% for average stride length and 2.23% for walking speed). In general, compared to previous studies, the INDIP method showed better or similar performances in the DMOs estimation based on a more complex validation design—both in terms of motor activities analyzed or motor gait impairments diversity—than what is currently being achieved.

### 4.5 INDIP usability in the real-world

Consistency of INDIP outputs was tested during 2.5 h of unsupervised acquisitions on the same participants involved in the laboratory experiments, while acceptability of the device, wearability and usability factors were also examined for the HYA participants (Appendix B). Regarding the 2.5 h real-world experiments, a wider range of WB durations was explored (from a minimum of 2.3 s to a maximum of 1741.6 s), which leads to WB lengths ranging from 0.49 m to 2,430.50 m. The minimum number of strides (four in all cohorts) was determined by the walking bout definition while a maximum value of 3,101 strides was observed for a PD patient. Extracted stride length values ranged from 0.19 m to 1.8 m (for definition the minimum stride length is 0.15 m) along with a very broad range of walking speeds from very slow (0.1 m/s) to very fast (1.6 m/s) and cadence values ranging from 44.69 steps/min (HOA) to 139.95 steps/min (CHF). It is important to notice that the values obtained for this subset of DMOs resulted to be consistent with those found in literature (Sofuwa et al., 2005; Panizzolo et al., 2014; Thingstad et al., 2015; Dujmovic et al., 2017; Iwakura et al., 2019). In general, during the 2.5 h acquisition, the INDIP resulted to be well accepted and no major technical or usability issues were declared.

### 4.6 Limitations and methodological choices

The findings of this study must be evaluated considering some limitations and specific methodological choices:

• The INDIP system in its full configuration requires sensors to be attached to the feet, shanks and lower trunk and sensor redundancy clearly limits wearability. For this reason, the INDIP is more suitable for a complete description of mobility performance rather than for long-term monitoring, for which a single-IMU solution is certainly preferable.
• The INDIP sensor redundancy was exploited for identifying gait events and detecting strides from pressure, inertial, and distance signals. For this study, it was decided to prioritize sensitivity to avoid missing events. However, stride detection specificity could be increased by selecting only strides identified by all the three types of sensors (i.e., gait events detected from both PI and foot mounted IMU, and non-zero DS measure during the stride interval).
• In this study, the distance sensors have not been properly integrated in a sensor fusion process. These sensors provide the inter-leg distance measure as further information (Bertuletti et al., 2018; Rossanigo et al., 2023), but the validation of this gait parameter was out of the scope of the present study.
• The PIs used are based on a low-cost technology (force sensitive resistor) with an expected lifetime of about 30 h, followed by an inevitable deterioration of the performances. Therefore, when the signal quality was no longer considered sufficient, PIs data was not used, and the trial was discarded from the here-presented analysis. The number of discarded acquisitions can be reduced ensuring the proper functionality of the adopted PIs before each data acquisition.
• The technical complexity associated to the implementation of multi-center experimental sessions and, in particular, problems related to the simultaneous use and synchronization of different technologies and sensors, the collection of a large number of trials in patients with mobility deficits and the presence of marker visibility issues led to discard about 13% of the participants’ data.
• Further analysis on INDIP outcomes could be performed to explore potential correlations between the results accuracy and the use of walking aids.

## 5 Conclusion

This work concerned the validation of a novel multi-sensor wearable system for digital mobility assessment in ecological environments. Its performance was evaluated based on a various and complex experimental protocol specifically designed for mobility assessment. Experiments included selected cohorts of participants with various conditions affecting gait characteristics performing a complex battery of motor tests designed to produce a heterogeneous and broad range of gait patterns. Results showed overall good/excellent reliability and high repeatability and accuracy for the DMOs analyzed across populations, walking speeds and walking bouts. The INDIP system is therefore a valuable candidate to collect reference standard data for the analysis of gait in real-world conditions.

## Data Availability

The datasets presented in this study can be found in online repositories. The names of the repository/repositories and accession number(s) can be found below: Data sample INDIP Validation Paper [Data set]. Zenodo. https://doi.org/10.5281/zenodo.7802795.
