# Supplementary material for: A multi-sensor wearable system for the assessment of diseased gait in real-world conditions
Source: Front Bioeng Biotechnol. 2023 Apr 21;11:1143248. doi: 10.3389/fbioe.2023.1143248 (PMC10194657; doi:10.3389/fbioe.2023.1143248)
Supplement: Supplementary file 1 [file DataSheet1.docx]

Supplementary Material

# Appendix A: INDIP hardware’s architecture and firmware

The main board is powered by a lithium polymers battery (nominal voltage: 3.7 V; capacity: 155 mAh, dimension: 24 mm L × 20 mm W × 3.8 mm H) scaled down to an operating voltage of 2.8 V by means of a high efficiency step-down converter. A form factor of 31 mm L × 29 mm W × 7 mm H (35 mm L × 47 mm W × 19 mm H including case and battery, with a mass of 16 g) has been achieved. The inertial module includes a 3D accelerometer and a 3D gyroscope and the full-scale ranges can be set differently according to the specific application (full-scale ranges of ±2/±4/±8/±16 g and ±125/±245/±500/±1000/±2000 dps). The analog front-end, which enables the connection with the pressure insole (cable length around 20 cm), consists of one negative feedback operational amplifier where a voltage divider with two resistors has been adopted as voltage reference. The Time-of-Flight infrared distance sensor, connected to the digital I/O port (cable length around 30 cm), includes a range sensor which can measure distances up to 0.2, 0.4, and 0.6 m at 50, 33, and 25 Hz, respectively. A form factor, including the case, of 36.2 mm L × 25.2 mm W × 11 mm H, with a mass of 4 g has been achieved.

The finite-state machine of the application firmware embedded on the main board of the INDIP system is depicted in Fig. A, whereas the description of the transitions between states is reported in Table A. The firmware has been implemented with the CubeMX hardware abstraction layer and the Atollic TrueSTUDIO® IDE for STM32. The main board is first programmed with a bootloader that enables the upload of the application firmware. The main advantage of the use of a bootloader is that if a new version of the application firmware is available (e.g., new available features, bug fixes, etc.) it can be easily uploaded on the microcontroller via USB. Once the application firmware has been correctly started a general check of the main components of the main board (i.e., inertial module, magnetometer, memory, BLE, battery charger, fuel gauge) and of the connected sensing peripherals (i.e., TOF, pressure insole) is carried out. If the check fails the system moves to the ERROR state and a red light goes on, otherwise the system moves from INIT to IDLE state. In this state all non-necessary components, except for the BLE module, are switched off to reduce power consumption. From the IDLE state, the main board can move to three different states depending on the event that occurs:

1. Start data logging (LOG state) on the on-board memory when the system receives the start LOG command either via USB, BLE, or external trigger (precondition: battery level >1% and free memory space >5%). The system returns to the IDLE state when it receives the STOP command either via USB, BLE, or external trigger or when the battery level reaches the 1% or when the system runs out of memory.
2. Start data streaming (TX state), i.e., transmit data via BLE, when the system receives the start TX command via BLE. This command is ignored in case the system is connected via USB. The system returns to the IDLE state when it receives the STOP command via BLE.
3. Start transmitting recorded data from memory (READOUT state) when the system receives the READOUT command with the specific recording to read. The system returns to the IDLE state when all data has been sent or when the read timeout has expired.

Finally, the system moves to STANDBY when the button has been pressed and held for at least 3s or the SHUTDOWN command has been sent by the user.


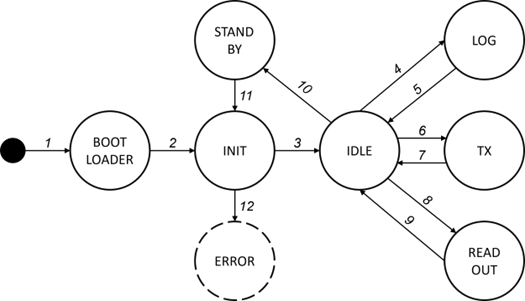


**Figure A.** Finite state machine of the main board of the INDIP system.

| **Transition** | **Current state** | **Event** | **Precondition** | **Action** |
| --- | --- | --- | --- | --- |
| 1 | - | Bootloader correctly uploaded | - | Go into BOOTLOADER |
| 2 | BOOTLOADER | - | Application firmware consistent | Go into INIT |
| 3 | INIT | Initialisation completed | No errors detected | Go into IDLE |
| 4 | IDLE | Start LOG via USB  Start LOG via BLE  Start LOG via external trigger | Battery level >1%; Memory (free space) >5%  Main board connected via BLE to a laptop or smartphone/tablet  USB connected (external trigger) | Go into LOG |
| 5 | LOG | Stop via USB  Stop via BLE  Stop via an external trigger  Memory full  Battery level <1% | USB connected  Main board connected via BLE to a laptop or smartphone/tablet USB connected (external trigger)  -  - | Go into IDLE |
| 6 | IDLE | Start TX via BLE | USB not connected | Go into TX |
| 7 | TX | Stop via BLE | - | Go into IDLE |
| 8 | IDLE | Read file via USB | - | Go into READOUT |
| 9 | READOUT | Read file completed or readout timeout | - | Go into IDLE |
| 10 | IDLE | Button pushed (>3s)  Shutdown via BLE | System on  Main board connected via BLE to a laptop or smartphone/tablet and not connected via USB | Go into STANDBY |
| 11 | STANDBY | USB connected  Button pushed (>3s) | -  System off | Go into INIT |
| 12 | INIT | - | System check-up failed | Go into ERROR |

**Table A.** Transition of the main board of the INDIP system

# Appendix B: INDIP Comfort Rating Scale

The Comfort Rating Scale included six statements for which the participant had to express their agreement with a number from 0 (low agreement) to 20 (high agreement) and, lastly, a final percentage score in the range 0-100% for the INDIP system was requested. The six statements included in the INDIP Comfort Rating Scale are reported in the following:

1. I was worried about how I looked when I was wearing the system – “*I was embarrassed to wear the system*”.
2. The system **did not** feel secure on my body – “*parts of the system were moving*”.
3. The system caused me some physical harm – “*it was painful to wear*”.

# Appendix B: INDIP Comfort Rating Scale

The Comfort Rating Scale included six statements for which the participant had to express their agreement with a number from 0 (low agreement) to 20 (high agreement) and, lastly, a final percentage score in the range 0-100% for the INDIP system was requested. The six statements included in the INDIP Comfort Rating Scale are reported in the following:

1. I was worried about how I looked when I was wearing the system – “*I was embarrassed to wear the system*”.
2. The system **did not** feel secure on my body – “*parts of the system were moving*”.
3. The system caused me some physical harm – “*it was painful to wear*”.
4. The system felt physically strange – “*I was physically aware of the system throughout testing*”.
5. The system affected the way I moved – “*the system inhibited or restricted my movement*”.
6. I **was not** happy wearing the system (for any of the reasons above or **other**).

The usability questionnaires were filled out by the HYA participating in the real-world experiments (n = 11/20). The preliminary results obtained suggested that the INDIP system was well accepted by the participants (average total score obtained by the sentences: 16/20) and considered suitable for its purpose (average final percentage score: 85%). However, further evidence from patients is required.
